# Allogenic Acellular Dermal Matrix and Xenogeneic Dermal Matrix as Connective Tissue Graft Substitutes for Long-Term Stability Gingival Recession Therapy: A Systematic Review and Meta-Analysis

**DOI:** 10.1055/s-0043-1772778

**Published:** 2023-10-17

**Authors:** Felita Clarissa Halim, Benso Sulijaya

**Affiliations:** 1 Periodontology Specialist Program, Department of Periodontology, Faculty of Dentistry, Universitas Indonesia, Jakarta, Indonesia; 2 Department of Periodontology, Faculty of Dentistry, Universitas Indonesia, Jakarta, Indonesia; 3 Dental Division, Universitas Indonesia Hospital, Depok, West Java, Indonesia

**Keywords:** allogeneic acellular dermal matrix, xenogeneic dermal matrix, connective tissue graft, gingival recession

## Abstract

Connective tissue graft (CTG) serves as a gold standard for gingival recession therapy. Yet the availability of CTG is limited, and it increases patient morbidity. Allogenic acellular dermal matrix (AADM) and xenogeneic dermal matrix (XDM) have been proven to be effective substitutes of CTG although the long-term stability is unclear. The aim of this study was to analyze the long-term stability outcome of gingival recession therapy using AADM and XDM compared to CTG. This study follows the Preferred Reporting Items for Systematic Reviews and Meta-Analyses (PRISMA) guidelines. Data were extracted independently from several online databases (PubMed, Scopus, and Embase). Five of 233 publications were included for final qualitative analysis and meta-analysis focusing on the mean difference of clinical parameters such as recession depth (RD), recession width (RW), probing depth (PD), clinical attachment loss (CAL), tissue thickness (TT), keratinized tissue width (KTW), and mean root coverage (MRC). Meta-analyses of RD, RW, CAL, TT, KTW, and MRC display an overall mean of 0.2 mm (95% confidence interval [CI]: –0.45 to –0.05), 0.29 mm (95% CI: –0.65 to 0.08), 0.2 mm (95% CI: –0.69 to 0.29), 0.25 mm (95% CI: –0.53 to 0.03), 0.26 mm (95% CI: –0.5 to 0.02), and 9.19% (95% CI: –13.95 to –4.43]), respectively, favoring the CTG. PD was the only parameter that favored the AADM or XDM with an overall mean of 0.03 mm (95% CI: –0.05 to 0.11). In all, if the long-term stability is the goal, the CTG is considered superior for gingival recession therapy. However, if it is contraindicated, the AADM and XDM might be considered as alternatives.

## Introduction

Gingival recession is a pathological migration of the gingival margin in an apical direction surpassing the cementoenamel junction that causes exposure of the root surface. 1 2 It affects a significant portion of the world population. A study in 2004 reported that incidence of gingival recession to be 89% in the population above the age of 20 years in Brazil. 3 This pathological condition has multifactorial etiology and predisposing factors such as plaque-induced inflammation, aggressive toothbrushing, periodontal disease, and both orthodontic and periodontal treatment. 1 4 5 Anatomical factors that may induce gingival recession are tooth anatomy and position, insufficient alveolar bone crest thickness, bone dehiscence, muscle traction, and frenulum anatomy. 6 Moreover, tissue phenotype is also considered another factor since patients with thin tissue phenotype have a higher risk of developing gingival recession. 4 The main concerns that are associated with gingival recessions are poor aesthetics and dentine hypersensitivity, although there are cases where patients are unaware of this condition and may not have any concerns. 7

There are a variety of techniques to treat gingival recession, providing long-term, stable, functional, and aesthetic root coverage with minimal morbidity. Coronally advanced flap (CAF) is the flap design of choice, but CAF alone might cause gingival recession relapse, especially in patients with thin, soft-tissue phenotype. 4 Autogenous connective tissue graft (CTG) is the gold standard 8 9 to treat gingival recession as it is most effective and predictable treatment to improve the percentage of root coverage, tissue thickness (TT), and the amount of keratinized tissue. 8 10 However, CTG comes with several disadvantages such as increased patient morbidity and surgical time, need for a second surgery site, and limited quantity. 11 According to a study in 2021, some patients seem to still remember the pain they experienced even a decade after the harvesting procedure, hence affecting their decision to accept therapy in the future. 12 It is not a surprise that substitutes of CTG are gaining in popularity as these may eliminate the disadvantages of CTG. 13

To avoid a second surgical site, there are a variety of biomaterials that may be used as substitutes such as allogenic acellular dermal matrix (AADM), xenogeneic dermal matrix (XDM), and enamel matrix derivative. 14 These materials can be used in addition to CAF, other flap designs, or even tunneling (TUN). 15

AADM was originally used to treat burn patients, but today it is used as a substitute to CTG in dental regenerative surgery without risk of rejection and disease transmission. 16 17 Clinical studies that use AADM reported increased keratinized tissue and increased root coverage. 18 19 AADM consists of an allogenic freeze dried connective tissue matrix, which has its epidermal layer and cellular components removed keeping its native three-dimensional structure composed of collagen and key extracellular matrix components including fibronectin, proteoglycans, and vascular channels, which support cell migration and capillary proliferation. Its allogeneic origin is restricted in most European countries; therefore, xenogeneic materials are more popular in countries with this restriction. 20

XDM is a porcine dermis–derived acellular collagen matrix consisting of three-dimensional type I/III collagen matrix and elastin. 21 22 Compared with AADM, XDM has greater availability and can be harvested in bigger quantities. 21 According to Lin et al, XDM provides a favorable environment for promoting migration, adhesion, and proliferation of periodontal ligament and oral fibroblasts cells. 23 When analyzed by scanning electron microscopy, this biomaterial shows a collagen arrangement with pores that allow vascularization and provide a framework for connective tissue cell migration. 16 In addition, the matrix thickness acts as a space maintainer favoring the formation of keratinized tissue. 24

Even though many clinical studies have been published, results often differ from one another, and long-term data are still scarce. Therefore, this systematic review focuses on comparing long-term root coverage results using CTG, AADM, and XDM.

## Methods

This systematic review was reported based on the Preferred Reporting Items for Systematic Review and Meta-Analyses (PRISMA) guideline and was registered in the International Prospective Register of Systematic Reviews (PROSPERO) under the code CRD42023444503.

### Focused Question

The purpose of this review was to compare AADM and XDM to CTG in the treatment of gingival recession. Focused question was set according to the population or Problem, Intervention, Comparison, and Outcome (PICO) framework applied as below:

- Population: healthy adult patients with gingival recession.
- Intervention: AADM or XDM as graft for treatment of recession.
- Comparison: use of CTG for treatment of recession.
- Outcome: clinical measurements such as recession depth (RD), recession width (RW), probing depth (PD), clinical attachment loss (CAL), TT, keratinized tissue width (KTW), percentage of complete root coverage (%CRC), and percentage of mean root coverage (%MRC).

### Search Strategy

Literature search using several databases, including PubMed, Embase, and Scopus, was performed to find articles from 2014 to March 2023. Keywords used for search in various combinations included “connective tissue graft,” xenogeneic, allogeneic, acellular, allograft, xenograft, dermis, “dermal matrix,” “gingival recession,” and “root coverage,” using AND/OR as Boolean operators.

### Inclusion and Exclusion Criteria

Articles with the following criteria were included in this review:

- Randomized controlled trials (RCT) evaluating the analyzed outcomes.
- Adult subjects with single or multiple gingival recessions treated with CTG compared with AADM or XDM, regardless of the surgical technique used in the study.
- Studies with at least 12 months of follow-up.

The exclusion criteria were the following:

- In vitro studies.
- Animal studies.
- Studies that use bone grafts or other material in combination with CTG, AADM, or XDM.
- Studies comparing different types of AADM or XDM and not to CTG.
- Studies comparing different surgical techniques.
- Studies on which clinical outcomes are irrelevant.

### Screening Method

Authors of this review (H.F.C. and S.B.) performed primary search on databases specified earlier and independently screened the titles and abstracts initially. Afterward, full-text articles were assessed to decide whether the articles met the inclusion criteria. Disagreements between reviewers were resolved through discussion until consensus was reached. Reasons for excluding studies were recorded.

### Data Extraction

Data from the included studies were extracted using an Excel spreadsheet (Microsoft, Redmond, WA, United States) created especially for this review. Data extracted were the following: title, author, year of publication, type of study, number of samples, surgical technique, follow-up, material used, and outcomes (RD, RW, PD, CAL, TT, KTW, %CRC, and %MRC) at baseline, follow-up, differences, and *p* -value comparing the differences between baseline and follow-up of each parameter of both materials used in the study.

### Risk-of-Bias Assessment

The risk of bias and quality of in RCTs were assessed by the Cochrane risk-of-bias 2.0 tool according to the Cochrane Handbook for Systematic Reviews of Interventions. 25 Evaluation was done by two reviewers and discrepancies were resolved through discussion.

### Statistical Analysis

A meta-analysis was performed to measure the overall effect (total weighted average) of each parameter comparing the controls and test groups of each RCT. The estimate was made using a random effects model with 95% confidence interval (CI). A meta-analysis was performed for parameters with data from at least two studies using Estimation Statistics with Confidence Intervals (ESCI) in Microsoft Excel for meta-analysis ( https://thenewstatistics.com/itns/esci/ ).

## Results

### Selection of Articles

**Fig. 1:**
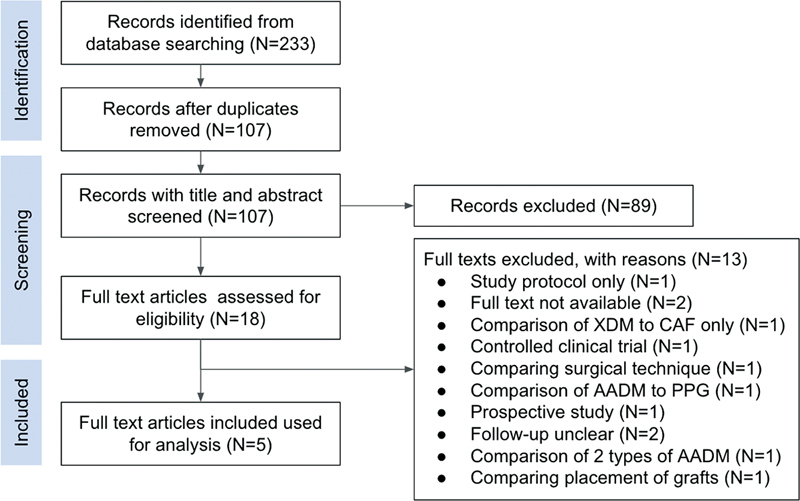
Preferred Reporting Items for Systematic Reviews and Meta-Analyses (PRISMA) flowchart.

Search results based on the PRISMA guidelines are depicted in Fig. 1 . A total of 233 articles were identified through the electronic search in three databases. After duplicates were removed, manual screening of 107 titles and abstract resulted in 89 articles being excluded and 18 full-text articles for further assessment. A total of five articles were used for final analysis. Excluded articles and reasons for exclusion are depicted in Fig. 1 .

Five RCTs were selected for the final analysis. 26 27 28 29 30 Table 1 shows the baseline information of included studies. Out of five studies, one compared CTG with AADM, while the other four studies compared CTG to XDM.

**Table 1:** Baseline information of selected studies

Sl no.	Study	Sample	Type of RCT	Country	Flap design	Follow-up (mo)	Graft material
1	Meza-Mauricio et al 26	41 patients and 130 recessions; 20 in control group (8 females, 12 males; age 38.1 ± 7.2 y), 21 in test group (9 females, 12 males; age: 36.3 ± 6.1 y)	Parallel, randomized, single-center controlled clinical trial	Brazil	CAF	12	CTG + XDM
2	Vincent-Bugnas et al 27	12 patients (8 females, 4 males); age: 23–55 y (mean: 41.2 ± 10.9 y); 74 recessions	Prospective single-center split-mouth randomized study	France	TUN	12	CTG + XDM
3	Gürlek et al 28	12 patients (8 females, 4 males); age: 31.41 ± 13.32 y, 82 recessions	Single-centered, split-mouth, randomized, controlled clinical trial	Turkey	MCAF	18	CTG + XDM
4	Rakasevic et al 29	20 patients (11 females, 9 males); mean age: 30.5 ± 7.9 y, 114 recessions	Split-mouth, single-center, prospective randomized controlled clinical trial	Serbia	MCAT	12	CTG + XDM
5	Barros et al 30	15 patients, 30 recessions	Parallel, randomized, single-center controlled clinical trial	Brazil	CAF	12	CTG + AADM

Abbreviations: AADM, allogenic acellular dermal matrix; CAF, coronally advanced flap; CTG, connective tissue graft; MCAF, modified coronally advanced flap; MCAT, modified coronally advanced tunnel; RCT, randomized controlled trial; TUN, tunnelling; XDM, xenogeneic dermal matrix.

### Risk-of-Bias Assessment

**Fig. 2:**
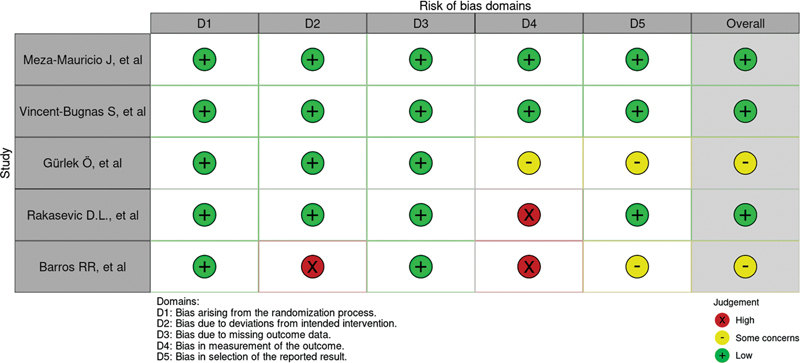
Quality evaluation of the randomized controlled trials (RCTs) using the RoB 2 tool (Cochrane Collaboration). 31 The risk of bias in the included studies was classified as either low (green), some concerns (yellow) or high (red).

**Fig. 3:**
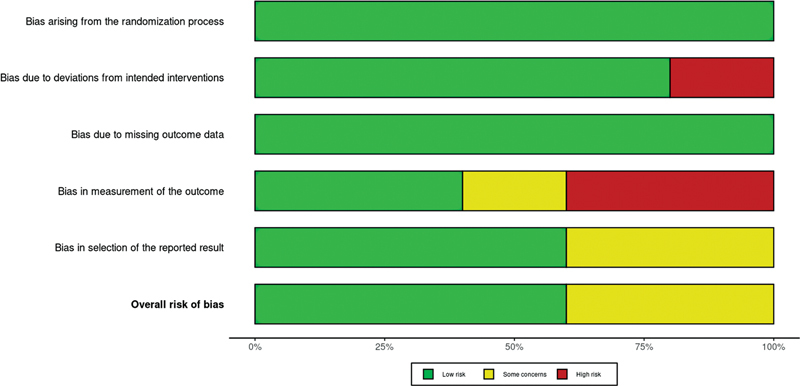
Summary of quality evaluation of the randomized controlled trials (RCTs) using the risk-of-bias (RoB) 2 tool (Cochrane collaboration). 31

The results of bias risk assessment for the included RCTs, using the Cochrane risk-of-bias tool, 31 are shown in Figs. 2 and 3 . Three articles had a low risk of bias, 26 27 29 and two were considered to have a moderate risk of bias. 28 30

### Quantitative Clinical Outcome of Included Studies

Quantitative outcomes from all included studies are summarized in Tables 2 and 3 . Quantitative data extracted include RD, RW, PD, CAL, TT, KTW, %CRC, and %MRC at baseline, follow-up, difference between baseline and follow up, and *p* -value for comparison of differences between controls and the test groups of each study.

**Table 2:** Quantitative outcomes of included studies

**Sl no.**	**Study**	**Flap design**	**Follow-up (mo)**	**Graft material**	**Outcome (mm)**											
					**Recession depth**				**Recession width**				**Probing depth**			
					**Baseline**	**Follow-up**	**∆ change**	***p-*** **value**	**Baseline**	**Follow-up**	**∆ change**	***p-*** **value**	**Baseline**	**Follow-up**	**∆ change**	***p-*** **value**
1	Meza-Mauricio et al 26	CAF	12	CTG	3.00 ± 0.78	0.24 ± 0.58	2.75 ± 0.11	0.03 a	4.36 ± 1.42	1.78 ± 2.20	2.58 ± 1.22	0.45 a	1.74 ± 0.47	2.22 ± 0.71	NA	NA
				XDM (mucoderm)	2.81 ± 0.77	0.42 ± 0.68	2.39 ± 0.12		4.45 ± 1.53	2.37 ± 2.30	2.08 ± 0.98		1.76 ± 0.55	2.06 ± 0.73	NA	
2	Vincent-Bugnas et al 27	TUN	12	CTG	2.9 ± 0.9	0.6 ± 0.74	2.3 ± 0.9	0.014 b	2.4 ± 0.7	0.7 ± 0.8	1.8 ± 0.8	0.048 b	1.9 ± 0.6	1.7 ± 0.5	0.2 ± 0.3	0.875
				XDM (mucoderm)	2.8 ± 1.0	1.0 ± 0.8	1.8 ± 0.8		2.6 ± 0.7	0.9 ± 0.8	1.7 ± 1.0		1.8 ± 0.5	1.6 ± 0.4	0.2 ± 0.3	
3	Gürlek et al 28	MCAF	18	CTG	2.60 ± 0.77	1.20 ± 0.33	2.50 ± 0.75	0.523	3.10 ± 0.71	0.22 ± 0.61	2.90 ± 1.00	0.04	1.80 ± 0.62	1.80 ± 0.56	0.05 ± 0.31	0.001
				XDM (mucoderm)	2.70 ± 1.00	0.32 ± 0.452	2.40 ± 0.92		3.10 ± 0.88	0.90 ± 1.50	2.20 ± 1.50		1.70 ± 0.66	2.00 ± 0.42	0.37 ± 0.49	
4	Rakasevic et al 29	MCAT	12	CTG	2.6 ± 1.23	0.32 ± 0.43	2.26 ± 1.17	0.569	2.44 ± 0.9	0.5 ± 0.52	1.95 ± 0.93	0.324	1.29 ± 0.46	1.13 ± 0.1	0.14 ± 0.03	0.919
				XDM (mucoderm)	2.9 ± 1.35	0.59 ± 0.8	2.31 ± 0.93		2.6 ± 1.1	0.43 ± 0.56	2.1 ± 1.05		1.27 ± 0.45	1.1 ± 0.33	0.16 ± 0.05	
5	Barros et al 30	CAF	12	CTG	3.15 ± 0.33	0.67 ± 0.61	2.48 ± 0.68	NS	NA	NA	NA	NA	1.31 ± 0.59	1.55 ± 0.70	0.24 ± 0.62	NS
				ADM (AlloDerm)	3.47 ± 0.42	0.93 ± 0.60	2.53 ± 0.54		NA	NA	NA		1.63 ± 0.73	1.77 ± 0.67	0.14 ± 0.70	
**Sl no.**	**Study**	**Flap design**	**Follow up (mo)**	**Graft material**	**Outcome (mm)**											
					**Clinical attachment loss**				**Tissue thickness**				**Keratinized tissue width**			
					**Baseline**	**Follow up**	**∆ change**	***p-*** **value**	**Baseline**	**Follow up**	**∆ change**	***p-*** **value**	**Baseline**	**Follow up**	**∆ change**	***p-*** **value**
1	Meza-Mauricio et al 26	CAF	12	CTG	4.56 ± 1.27	2.89 ± 1.22	NA	NA	0.85 ± 0.25	1.53 ± 0.38	0.77 ± 0.05	0.01 a	2.42 ± 1.29	3.34 ± 1.11	0.99 ± 1.23	0.06
				XDM (mucoderm)	4.14 ± 0.99	2.65 ± 0.97	NA	NA	0.81 ± 0.23	1.26 ± 0.22	0.54 ± 0.03		2.43 ± 1.12	3.06 ± 0.92	0.63 ± 0.83	
2	Vincent-Bugnas et al 27	TUN	12	CTG	4.8 ± 1.0	2.3 ± 0.8	2.5 ± 0.9	<0.001 b	0.8 ± 0.3	1.9 ± 0.3	1.0 ± 0.3	<0.001 b	2.2 ± 1.3	3.0 ± 1.0	0.7 ± 0.8	0.19		
				XDM (mucoderm)	4.6 ± 1.2	2.6 ± 0.9	2.0 ± 0.9		0.8 ± 0.2	1.2 ± 0.2	0.4 ± 0.2		2.1 ± 1.6	2.5 ± 1.2	0.4 ± 0.7			
3	Gürlek et al 28	MCAF	18	CTG	4.40 ± 1.00	0.49 ± .098	3.90 ± 1.10	0.362	NA	NA	NA	NA	3.70 ± 1.10	4.20 ± 0.98	0.51 ± 0.60	0.088
				XDM (mucoderm)	4.40 ± 1.10	0.71 ± 1.30	3.70 ± 1.60		NA	NA	NA		3.40 ± 1.20	3.70 ± 0.93	0.32 ± 0.52	
4	Rakasevic et al 29	MCAT	12	CTG	3.86 ± 1.32	0.88 ± 0.92	2.98 ± 1.40	0.48	0.69 ± 0.26	1.3 ± 0.38	0.7 ± 0.34	0.045 b	2.43 ± 1.4	3.27 ± 1.03	0.84 ± 1	0.967
				XDM (mucoderm)	4.09 ± 1.4	0.92 ± 1.2	3.17 ± 1.25		0.61 ± 0.2	1.39 ± 0.44	0.78 ± 0.32		2.44 ± 1.3	3.28 ± 0.9	0.85 ± 1.2	
5	Barros et al 30	CAF	12	CTG	NA	NA	NA	NA	NA	NA	NA	NA	2.05 ± 0.78	3.20 ± 1.01	NA	NS
				ADM (AlloDerm)	NA	NA	NA		NA	NA	NA		1.90 ± 0.54	3.20 ± 0.77	NA	

Abbreviations: AADM, allogeneic acellular dermal graft; CAF, coronally advanced flap; CTG, connective tissue graft; MCAF, modified coronally advanced flap; MCAT, modified coronally advanced tunneling; NA, not available; NS, not significant; TUN, tunneling; XDM, xenogeneic dermal graft.

a Statistically significant difference between the groups by Student's *t* -test or Fisher's exact test ( *p* < 0.05).

b Statistically significant difference between the groups by Wilcoxon signed-rank test ( *p* < 0.05).

**Table 3:** Complete root coverage and mean root coverage result in included RCTs

Sl no.	Study	Flap design	Follow-up (mo)	Graft material	Outcome			
					% complete root coverage	*p* -value	% mean root coverage	*p* -value
1	Meza-Mauricio et al 26	CAF	12	CTG	83.3	0.01 a	91.79 ± 10.1	0.06
				XDM (mucoderm)	70.3		80.19 ± 16.3	
2	Vincent-Bugnas et al 27	TUN	12	CTG	48.7 ± 6.8	NA	80.6 ± 23.7	0.005 b
				XDM (mucoderm)	24.3 ± 8.2		68.8 ± 23.4	
3	Gürlek et al 28	MCAF	18	CTG	87.8	NA	NA	NA
				XDM (mucoderm)	70.7		NA	
4	Rakasevic et al 29	MCAT	12	CTG	51.9	0.584	87.6 ± 15.1	0.48
				XDM (mucoderm)	46.8		85.25 ± 14.9	
5	Barros et al 30	CAF	12	CTG	NA	NA	NA	NA
				ADM (AlloDerm)	NA		NA	

Abbreviations: AADM, allogeneic acellular dermal graft; CAF, coronally advanced flap; CTG, connective tissue graft; MCAF, modified coronally advanced flap; MCAT, modified coronally advanced tunneling; NA, not available; RCT, randomized controlled trial; TUN, tunneling; XDM, xenogeneic dermal graft.

a Statistically significant difference between the groups by Student's *t* -test or Fisher's exact test ( *p* < 0.05).

b Statistically significant difference between the groups by Wilcoxon signed-rank rest ( *p* < 0.05).

### Meta-Analysis

**Fig. 4:**
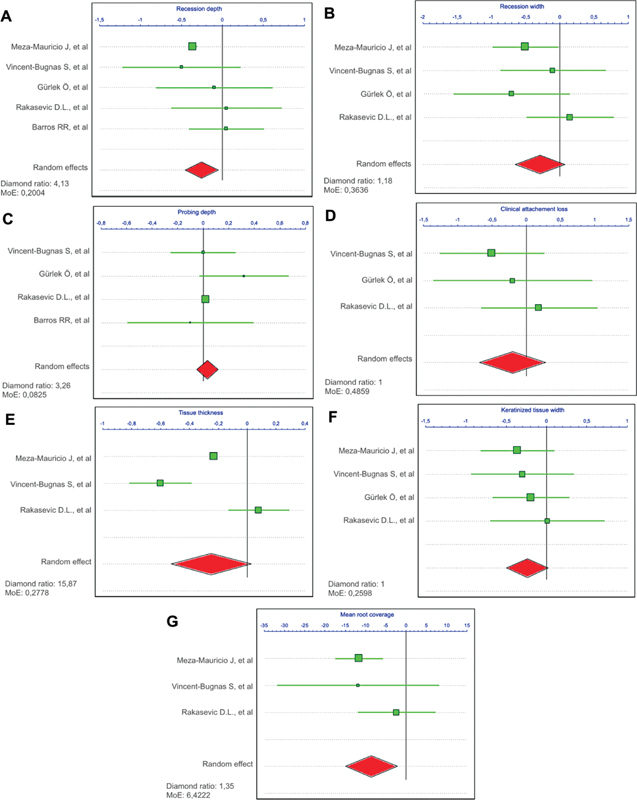
Forest plot for connective tissue graft (CTG; control group) versus xenogeneic dermal matrix/allogenic acellular dermal matrix (XDM/AADM; test group) when comparing the difference as baseline to follow-up of ( **A** ) recession depth, ( **B** ) recession width, ( **C** ) probing depth, ( **D** ) clinical attachment loss, ( **E** ) tissue thickness, ( **F** ) keratinized tissue width, and ( **G** ) mean root coverage. The weighted means are presented with 95% confidence interval (CI).

A meta-analysis was performed to compare the mean RD, RW, PD, CAL, TT, KTW, and MRC at follow-up in gingival recessions treated with CTG and AADM or XDM as shown in Fig. 4 . A meta-analysis of RD was conducted using data from all included studies, which resulted in a mean difference of 0.25 mm (95% CI: –0.45 to –0.05). As for RW, data from four studies were included and results showed a mean difference of 0.29 mm (95% CI: –0.65 to 0.08). A meta-analysis of PD used data from four studies and results showed a mean difference of 0.03 mm (95% CI: –0.05 to 0.11]). A meta-analysis of CAL included data from three studies and results showed a mean difference of 0.2 mm (95% CI: –0.69 to 0.29). A mean difference of 0.25 mm (95% CI: –0.53 to 0.03) was reported for TT with data from three studies. A meta-analysis of KTW used data from four included studies, which resulted in a mean difference of 0.26 mm (95% CI: –0.5 to 0.02). Finally, a meta-analysis of MRC was performed using data from three studies and results showed a mean difference of 9.19% (95% CI: –13.95 to –4,43). Almost all parameters reported results in favor of CTG compared with its alternative, except for PD.

## Discussion

With the increasing prevalence of gingival recession, finding treatment options and alternatives have become a priority in periodontal practice. 32 Alternatives to the gold standard, CTG, have been used for years and have been reported to be a safe alternative that provides regeneration of gingival tissue and promotes wound healing. 33 34 However, studies with long-term results are still very scarce. The main objective of this review was to compare the long-term results of CTG substitutes such as AADM and XDM. The paucity of long-term studies was proven by an electronic search from the last decade resulting in only five studies meeting the inclusion criteria, with only one of the five included studies comparing AADM to CTG. This could be because the use of allogeneic origin material is restricted in most European countries. 20

Meza-Mauricio et al 26 reported better results for all parameters in favor of CTG compared with XDM at 1 year of follow-up. In the CAF + XDM group, some parameters showed a higher value at 6 months of follow-up, which then reported a slight decrease at the 12-month follow-up. The mean gingival thickness gain, CRC, and recession reduction were all significantly lower in the CAF + XCM group. However, the other parameters, although in favor of the CAF + CTG group, showed no significant difference between both groups at 1 year. Some of the advantages of using a CTG substitute reported were less surgical time and less pain. Surgical time needed was 48.8 ± 15.06 and 36 ± 8.1 minutes for CTG and XDM, respectively. Patients in the test group experienced significantly less pain within the first 7 days, but overall significant improvement in quality of life was reported for both groups without a significant difference between groups.

In a study by Vincent-Bugnas et al, 27 differences in RD, RW, CAL, and TT were statistically significant in favor of the control group. However, both XDM and CTG showed significant differences between baseline and follow-up. The superiority of XDM reported was similar to previous studies: reduced postoperative pain intensity in the first week after surgery.

Rakasevic et al 29 also reported significant improvement for both groups from baseline to 12 months postoperatively, but no significant difference was found between groups during follow-up. There was no statistically significant difference in CRC and MRC between the treatment modalities 6 and 12 months postoperatively within the groups, but it was statistically significant between the groups (2.96 ± 11.8 in the control group and –1.71 ± 13.7% in the test group). Twice as many patients presented a complete coverage of all recessions in the control group than in the test group after 12 months.

An 18-month follow-up study by Gürlek et al 28 reported similar results, favoring control groups using CTG compared with XDM. This shows that regardless of the technique used, long-term results differ when different materials are used to treat gingival recession.

In the only study that compared CTG to AADM, Barros et al 30 reported no significant difference between both control and test groups for all the measured parameters. In fact, at 12 months, results were slightly in favor of gingival recession treated with AADM. The result of this review is in accordance with a review by Zhang et al 35 that included trials with less than a year of follow-up. The ADM treatment for patients with gingival recession may be superior to CTG in gaining CAL, but CTG has a significant advantage over ADM for gaining KTW. Tavelli et al 36 reported a significant relapse at 12 years of follow-up when gingival recessions were treated with AADM regardless of the flap design used. Predictors of long-term stability may be determined from KTW at baseline and TT at 6 months of follow-up.

Modifications to some substitutes have also been studied. Tavelli et al 34 evaluated the efficacy of recombinant human platelet–derived growth factor BB (rhPDGF-BB) combined with a cross-linked xenogeneic (porcine) collagen matrix (XCM) for the treatment of multiple adjacent gingival recessions. In this study, the test group was XCM soaked in rhPDGF-BB, while the control group used saline with the collagen matrix. Results showed that rhPDGF enhances the 6-month root coverage outcomes of a xenogeneic collagen matrix. Increased volumetric and aesthetic outcomes were also observed in the sites that received rhPDGF. The use of the growth factor promoted a faster recovery and less postoperative morbidity during the first 5 days, while the other investigated patient-reported outcomes were similar between the two groups. Future studies are needed to investigate the long-term results and cost-effectiveness of rhPDGF-BB when utilized with a collagen scaffold for root coverage procedure compared with CTG.

As shown in Table 4 , results from the meta-analyses of several outcomes in selected studies, after at least 1 year, still show the superiority of CTG compared with other soft-tissue graft materials. It was also observed that several flap designs were used in the included studies from CAF, TUN, modified coronally advanced flap (MCAF), and modified coronally advanced tunnel (MCAT). To our knowledge, the CAF technique is the most documented approach in the literature, and in combination with CTG, it is the gold standard in the treatment of gingival recession. 37 Moreover, modified CAF, without vertical releasing incision, has shown high success rates in treating multiple gingival recessions. 38 Drawbacks such as insufficient amount of KTW, noncarious cervical lesion or reduced vestibule depth indicate the need for a different surgical approach besides the CAF or MCAF technique. 39 Comparing the different techniques, quantitative data reported higher increases in RD, RW, %CRC, and %MRC for the CAF and MCAF techniques. TUN and MCAT are superior in increasing KTW. It is reported that CAF is more effective than TUN when it comes to root coverage, when the same grafts were used in both techniques, regardless of its origin. 40 41 42

**Table 4:** Data of RD, RW, PD, CAL, TT, KT, and MRC at follow-up selected for meta-analysis

Study	CTG			AADM or XDM			Weight (%)	WMD (95%CI)	Year
	Mean	SD	*N*	Mean	SD	*N*		*N*	
**Recession depth**									
Meza-Mauricio et al 26	2.75	0.11	42	2.39	0.12	42	24.5	–0.36 (–0.41 to –0.31)	2021
Vincent-Bugnas et al 27	2.3	0.9	12	1.8	0.8	12	17.9	–0.5 (–1.22 to –0.22)	2020
Gürlek et al 28	2.5	0.75	12	0.32	0.92	12	18.0	–2.18 (–2.89 to –1.47)	2020
Rakasevic et al 29	2.26	1.17	20	2.31	0.93	20	18.3	0.05 (–0.63 to 0.73)	2020
Barros et al 30	2.48	0.68	15	2.53	0.54	15	21.3	0.05 (–0.41 to 0.51)	2015
							100	–0.25 (–0.45 to –0.05)	
**Recession width**									
Meza-Mauricio et al 26	2.58	1.22	42	2.08	0.98	42	37.0	–0.5 (–0.98 to –0.02)	2021
Vincent-Bugnas et al 27	1.8	0.8	12	1.7	1	12	20.1	–0.1 (–0.87 to 0.67)	2020
Gürlek et al 28	2.9	1	12	2.2	1	12	17.1	–0.7 (–1.55 to 0.15)	2020
Rakasevic et al 29	1.95	0.93	20	2.1	1.05	20	25.9	0.15 (–0.49 to 0.78)	2020
							100	–0.29 (–0.65 to 0.08)	
**Probing depth**									
Vincent-Bugnas et al 27	0.2	0.3	12	0.2	0.3	12	10.4	0 (–0.25 to 0.25)	2020
Gürlek et al 28	0.05	0.31	12	0.37	0.49	12	5.9	0.32 (–0.03 to 0.67)	2020
Rakasevic et al 29	0.14	0.03	20	0.16	0.05	20	80.8	0.02 (–0.01 to 0.05)	2020
Barros et al 30	0.24	0.62	15	0.14	0.7	15	2.9	–0.1 (–0.59 to 0.39)	2015
							100	0.03 (–0.05 to 0.11)	
**Clinical attachment loss**									
Vincent-Bugnas et al 27	2.5	0.9	12	2	0.9	12	45.5	–0.5 (–1.26 to 0.26)	2020
Gürlek et al 28	3.9	1.1	12	3.7	1.6	12	19.6	–0.2 (–1.36 to 0.96)	2020
Rakasevic et al 29	2.98	1.4	20	3.17	1.25	20	34.9	0.19 (–0.66 to 1.04)	2020
							100	–0.2 (–0.69 to 0.29)	
**Tissue thickness**									
Meza-Mauricio et al 26	0.77	0.05	42	0.54	0.03	42	37.5	–0.23 (–0.25 to –0.21)	2021
Vincent-Bugnas et al 27	1	0.3	12	0.4	0.2	12	31.3	–0.6 (–0.82 to –0.38)	2020
Rakasevic et al 29	0.7	0.34	20	0.78	0.32	20	31.2	0.08 (–0.13 to 0.29)	2020
							100	–0.25 (–0.53 to 0.03)	
**Keratinized tissue width**									
Meza-Mauricio et al 26	0.99	1.23	42	0.63	0.83	42	33.5	–0.36 (–0.82 to 0.10)	2021
Vincent-Bugnas et al 27	0.7	0.8	12	0.4	0.7	12	18.7	–0.3 (–0.94 to 0.34)	2020
Gürlek et al 28	0.51	0.6	12	0.32	0.52	12	33.4	–0.19 (–0.67 to 0.28)	2020
Rakasevic et al 29	0.84	1	20	0.85	1.2	20	14.4	0.01 (–0.7 to 0.72)	2020
							100	–0.26 (–0.5 to 0.02)	
**Mean root coverage**									
Meza-Mauricio et al 26	91.79	10.1	42	80.19	16.3	42	67.4	–11.6 (–17.48 to –5.71)	2021
Vincent-Bugnas et al 27	80.6	23.7	12	68.8	23.4	12	6.4	–11.8 (–31.74 to 8.14)	2020
Rakasevic et al 29	87.6	15.1	20	85.25	14.9	20	26.2	–2.35 (–11.95 to 7.25)	2020
							100	–9.19 (–13.95 to –4.43)	

Abbreviations: AADM, allogenic acellular dermal matrix; CAL, clinical attachment loss; CTG, connective tissue graft; KT, keratinized tissue; MRC, mean root coverage; PD, probing depth; RD, recession depth; RW, recession width; SD, standard deviation; TT, tissue thickness; WMD, weighted mean difference; XDM, xenogeneic dermal matrix.

Several systematic reviews have assessed the effectiveness of CTG substitutes; however, to our knowledge, most systematic reviews consist of studies with less than a year of follow-up. 35 43 44 All the studies reported that both XDM and AADM are suitable alternatives with promising short-term results.

This result is similar when it is applied to modifying soft tissue around dental implants. CTG and its substitutes resulted in increased TT, but significant difference favoring CTG is reported in three of seven studies included in this review. CTG is also considered the gold standard for soft-tissue augmentation around dental implants. 45

## Conclusion

Within the limitations of the present study, it can be concluded that CTG still shows better long-term (12- to 18-month) results compared with AADM or XDM, regardless of the flap design or surgical technique. However, when CTG harvesting is not indicated, AADM or XDM, depending on availability, may be a good alternative in treating gingival recession. Further studies with longer follow-up are needed to determine the long-term stability of grafts with xenogeneic and allogeneic origins.
